# Braithwaite’s Retrospect of Practical Medicine and Surgery

**Published:** 1863

**Authors:** 


BRAITHWAITE'S RETROSPECT OF PRACTICAL MEDICINE AND

SURGERY continues to be published with all its original good qualites, by W. A. TOWNSEND, 39 Walker Street, New York.

